# Systemic Lupus Erythematosus Presenting as Optic Neuropathy: A Case Report

**DOI:** 10.7759/cureus.4806

**Published:** 2019-06-03

**Authors:** Salman Zahid, Mustafa Iqbal

**Affiliations:** 1 Internal Medicine, Hayatabad Medical Complex, Peshawar, PAK; 2 Ophthalmology, Khyber Teaching Hospital, Peshawar, PAK

**Keywords:** : systemic lupus erythematosus, optic neuropathy, optic neuritis

## Abstract

Systemic lupus erythematosus (SLE) is a chronic inflammatory disease with a wide variety of clinical presentations as a result of its effect on several organ systems. Optic nerve involvement in SLE is very uncommon, but optic neuritis can be the initial manifestation of SLE. A previously healthy 47-year-old woman developed blurring of vision in the left eye for the last three weeks and associated periorbital pain that worsened with eye movement. On a review of systems, she reported a photosensitivity rash, painless oral ulcers, and generalized arthralgia. On examination, she had relative afferent pupillary defect (RAPD) in the left eye. A visual field analysis revealed bilateral arcuate defects. Positive antinuclear antibodies (ANA), anti-beta2-glycoprotein I, and low complement levels of C4 were found consistent with the diagnosis of SLE. We present a case of optic neuropathy as the initial manifestation of SLE in a 47-year-old lady and an associated review of the literature.

## Introduction

Systemic lupus erythematosus is a chronic autoimmune inflammatory disease with a wide variety of clinical presentations as a result of its effect on several organ systems. The etiopathogenesis of SLE is complex, but it is believed to be due to a complex interaction between genetic, hormonal, and environmental factors. The disease exhibits its symptoms due to the formation of autoantibodies, immune complex deposition, and complement activation [1]. The incidence of SLE is one to 10 per 100,000 person-years with a prevalence of 20-200 per 100,000 person-years. The disease mainly affects women of child-bearing age. It has been reported that the incidence of SLE is higher in Asia, African, and Hispanic population [2]. Optic nerve involvement in SLE is very uncommon but optic neuritis can be the initial manifestation of SLE. Central nervous system (CNS) involvement occurs in 20%-40% of patients whereas only 1% of patients have optic nerve or optic chasm involvement. The pathogeneses of SLE optic neuritis involves occlusive vasculitis affecting the small arterioles of the optic nerve, leading to axonal necrosis. It is of paramount importance that ophthalmologists distinguish between SLE optic neuritis and idiopathic optic neuritis since visual impairment and treatment dependence is stronger with SLE-related optic neuritis [3].

We present a case of optic neuropathy as the initial manifestation of SLE in a 47-year-old lady, with an associated review of the literature.

## Case presentation

A 47-year-old woman presented with blurring of vision in the left eye for the last three weeks, associated with periorbital pain that worsened with eye movement. On a review of systems, she reported a photosensitivity rash, generalized arthralgia, painless oral ulcers, exertional dyspnea, exertional chest pain, and generalized weakness. Past medical history was significant for iron deficiency anemia secondary to menorrhagia for which she was taking iron supplements. She was a mother of two children with no history of miscarriages. The rest of the personal and family history was unremarkable.

**Figure 1:**
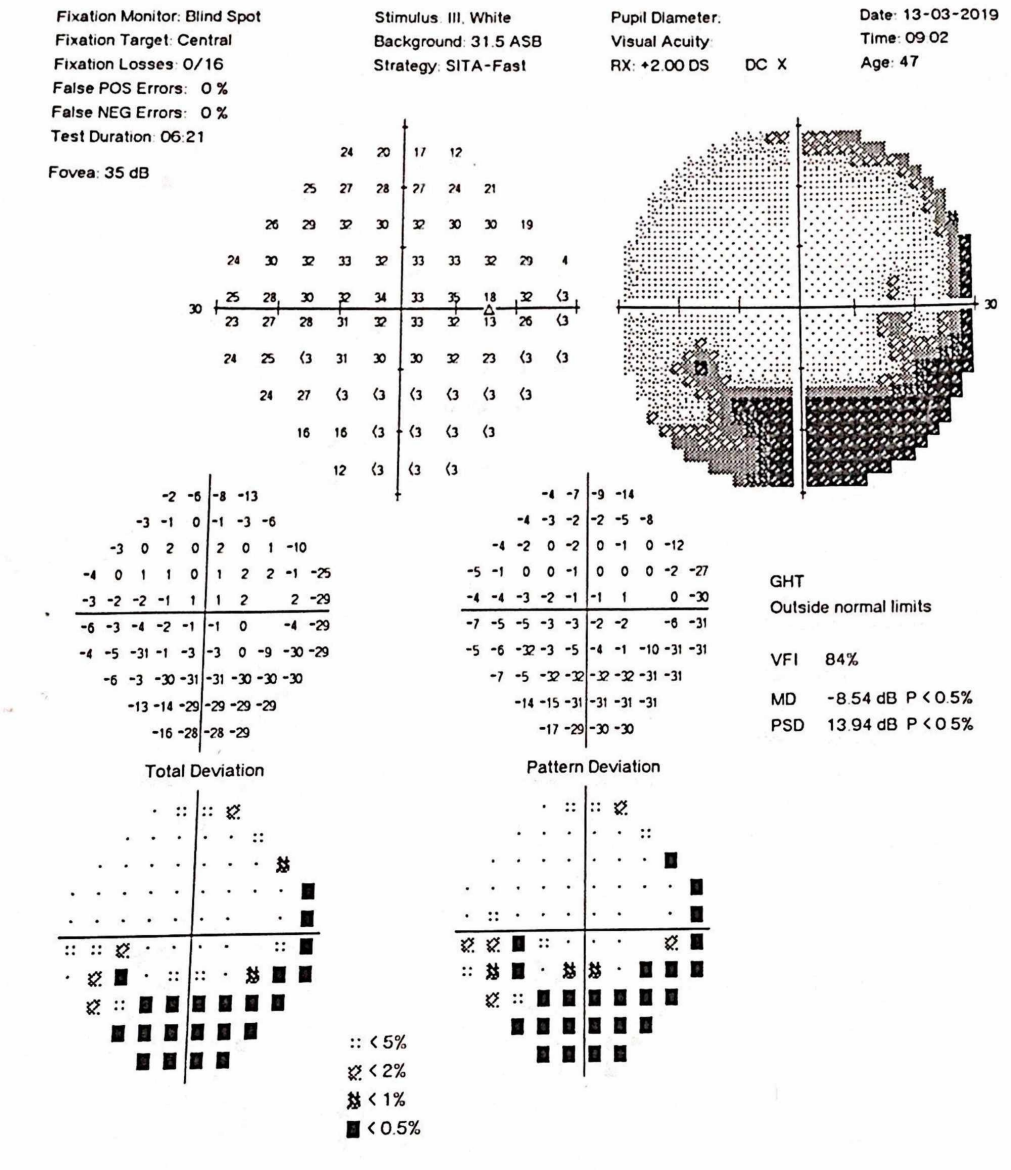
Visual Field Right Eye

**Figure 2:**
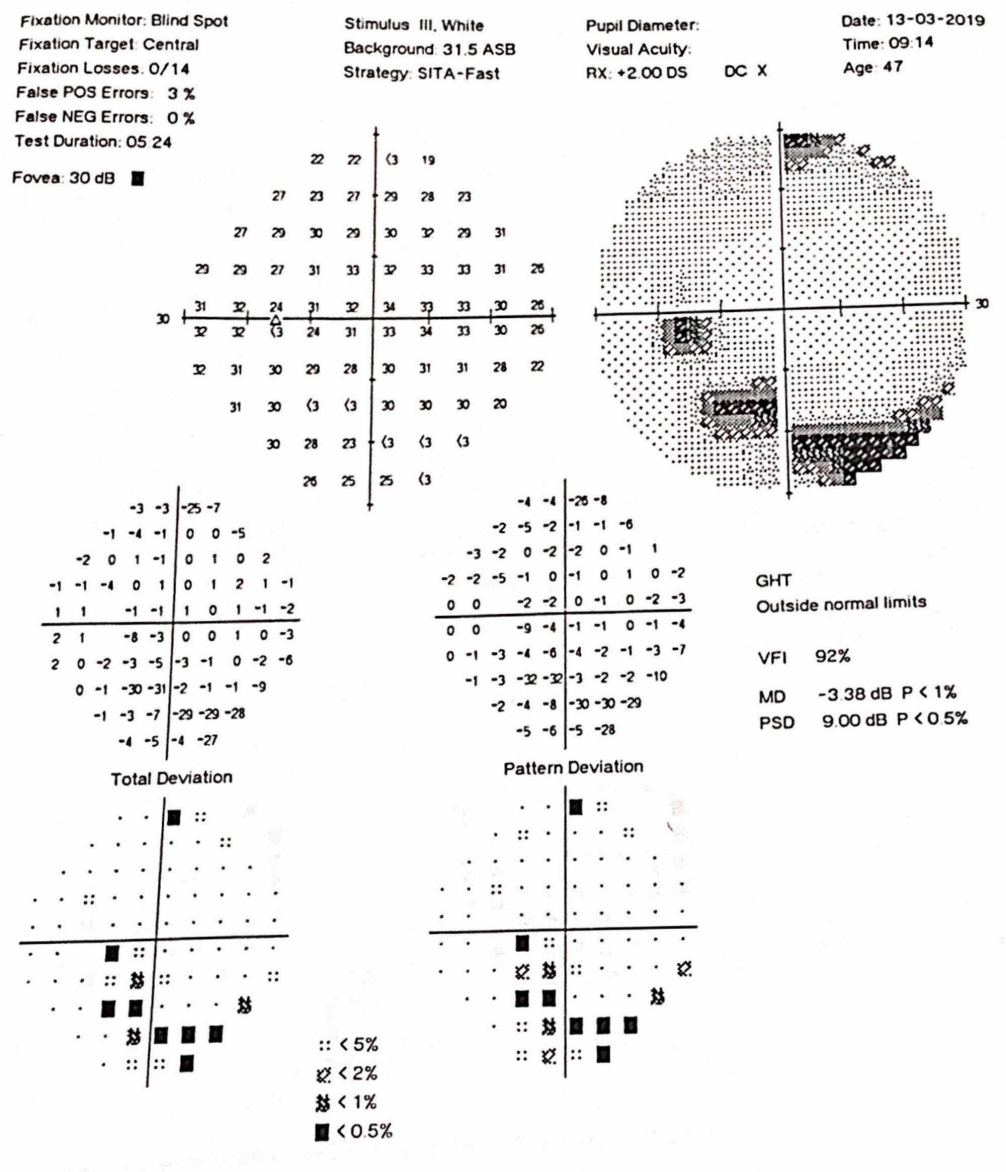
Visual Field Left Eye

On examination, visual acuity of the right eye was 6/6 and in the left eye, it was 6/9 (no improvement with pinhole). Relative afferent pupillary defect (RAPD) could be observed in the left eye. Intraocular pressure was 15 mmHg bilaterally. The anterior chamber was quiet and the fundus appeared normal, with no evidence of optic disk swelling. A visual field analysis revealed inferior arcuate defects bilaterally (Figures 1-2).

All basic laboratory tests were normal, apart from hemoglobin (7 mg/dl), peripheral smear (microcytic hypochromic anemia), erythrocyte sedimentation rate (ESR; 50 mm), and C-reactive protein (CRP; 18 mg/dl, n<5 mg/dl). The autoimmune profile revealed positive antinuclear antibodies (ANA) (1+ speckled), positive immunoglobulin M (IgM), anti-beta2-glycoprotein I (35 RU/ml, n<20 RU/ml), and low levels of complement C4 (0.08 G/L, n>0.15 G/L). Moreover, the patient had a positive anti-parietal cell antibody along with low serum vitamin B12 levels (40 ng/ml, n>200 ng/ml).

Magnetic resonance imaging (MRI) of the brain and spine was normal, except for an incidental finding of dorsal subarachnoid web indenting the spinal cord. Based on the history, the physical exam finding of RAPD, and lab findings, the patient was diagnosed as a case of systemic lupus erythematosus manifesting as optic neuritis. The patient was started on methylprednisolone 1 gm I/V pulse for three days followed by oral prednisone 1 mg/kg/day and hydroxychloroquine 200 mg OD. Injection vitamin B12 was also administered Intramuscularly.

On follow-up after two weeks, the patient reported significant subjective improvement in symptoms. Visual acuity in the left eye and right eye were 6/6. On pupillary exam, left RAPD was still present. Visual field analysis showed that the visual field defects had resolved. Moreover, ESR and CRP trended down and hemoglobin improved from 7.5 mg/dl to 9.1 mg/dl.

Relapse of symptoms occurred after four weeks when the dose of oral prednisone was tapered down to 30 mg. Prednisone dose was increased to 60 mg, and the patient responded well to treatment. Azathioprine 50 mg OD was started after measuring the thiopurine methyltransferase (TMPT) activity. TMPT activity was 22.5 U/ml red blood cell (RBC) (normal range is 15.1-26.4U/ml RBC). White blood cell (WBC) counts were measured weekly and a target of WBC count between 6000 and 8000/mm^3^ was set. Over a period of three weeks, the azathioprine dose was increased to 150 mg as steroids were slowly tapered off.

## Discussion

Neuro-ophthalmic manifestations are rare in SLE, with a prevalence of 3.6%. Among the neuro-ophthalmic manifestations, the most common is optic neuritis, which is followed by myasthenia gravis, visual field defects, and pseudotumor cerebri [4]. SLE is a rare disease and neuropsychiatric manifestations of SLE are even rarer. According to the American College of Rheumatology's (ACR's) proposed criteria for neuro-psychiatric SLE (NPSLE), there are 12 syndromes involving the CNS. Based on the ACR's proposed nomenclature, isolated optic neuritis should be classified as cranial neuropathy, which best fits our case [5].

The pathogenesis of optic neuritis caused by SLE differs from neuromyelitis optica (NMO) and multiple sclerosis (MS). SLE-associated optic neuropathy is because of vasculitis or thrombotic vaso-occlusive phenomenon involving small arterioles, leading to axonal necrosis. The poor visual outcome in cases of SLE optic neuropathy is due to the permanent damage caused by occlusive vasculitis. In comparison, NMO is an inflammatory demyelinating disease in which the primary target of antibodies is the aquaporin, or AQP, channels of astrocytes. MS is also a demyelinating disease, with oligodendrocytes as the primary target. The difference in the etiopathogenesis can explain the difference in the initial presentation, response to treatment, and prognosis [3,6].

The first case of SLE manifesting as optic neuropathy was reported in 1978 by Cinfro and Frenkel [7]. Fourteen more cases have been reported since the publication of the first case of SLE presenting as optic neuropathy [7-12]. A review of 13 cases by Fridge M et al. reported that in 31% of cases, the diagnosis of SLE was preceded by optic neuropathy. In our case, the diagnosis of optic neuropathy was concurrent with the diagnosis of SLE. On fundoscopy, 76.9% of patients had optic disk swelling. Regarding treatment, 69.2% were treated with methylprednisolone pulse therapy, 92.3% with high-dose oral corticosteroids (1 mg/kg/day), 15.4% patients received monthly intravenous (IV) pulses of cyclophosphamide, and 15.4% patients received anticoagulation. In terms of the outcome, visual recovery was partial in 17 eyes (77.3%) and complete visual recovery was seen in 22.7% cases. [11]. The case we report did not show any optic disk swelling or magnetic resonance imaging (MRI) changes and the visual recovery was complete. A recently reported case by Amigo et al. described superior arcuate defects in the visual field and the unique feature was that SLE was diagnosed seven months after the first manifestation of the disease as optic neuropathy. In our case, inferior arcuate defects in the visual fields could be appreciated [12].

The recommended treatment for SLE optic neuritis is high-dose intravenous methylprednisolone (1 g/day for three days) followed by oral prednisone (1 mg/kg/day) [13]. One study found that 29% of patients may be refractory to steroids. In such patients, IV pulse-dosed cyclophosphamide for six months has been studied in open clinical trials and has proven to be effective in cases of steroid-refractory optic neuropathy [14]. Moreover, hydroxychloroquine may play a role in preventing thrombosis in patients having a concurrent anti-phospholipid syndrome. It may also help to prevent thrombosis of the small vessels supplying the optic nerve [15]. We can also consider warfarin or aspirin in cases of antiphospholipid syndrome causing optic neuropathy in SLE [16]. The optic neuropathy associated with SLE has an excellent response to treatment, provided that therapy is instituted early in the disease's course [4].

## Conclusions

Optic neuropathy as the initial manifestation of SLE is extremely rare, but it can present as progressive vision loss, which is reversible with appropriate treatment. At presentation, the only objective finding of optic neuropathy could be a very subtle relative afferent pupillary defect (RAPD). Hence, a good history and thorough physical exam are of paramount importance in making the proper diagnosis. Timely identification of the disease can be sight-saving, as the early institution of treatment has an excellent prognosis.

## References

[1] Maidhof W, Hilas O (2012). Lupus: an overview of the disease and management options. P & T.

[2] Ali A, Sayyed Z, Ameer MA (2018). Systemic lupus erythematosus: an overview of the disease pathology and its management [Retracted]. Cureus.

[3] Lin YC, Wang AG, Yen MY (2009). Systemic lupus erythematosus-associated optic neuritis: clinical experience and literature review. Acta Ophthalmol.

[4] Man BL, Mok CC, Fu YP (2014). Neuro-ophthalmologic manifestations of systemic lupus erythematosus: a systematic review. Int J Rheum Dis.

[5] ACR Ad Hoc Committee on Neuropsychiatric Lupus Nomenclature (1999). The American College of Rheumatology nomenclature and case definitions for neuropsychiatric lupus syndromes. Arthritis Rheum.

[6] Adawi M, Bisharat B, Bowirrat A (2014). Systemic lupus erythematosus (SLE) complicated by neuromyelitis optica (NMO - Devic's disease): clinic-pathological report and review of the literature. Clin Med Insights Case Rep.

[7] Cinefro RJ, Frenkel M (1978). Systemic lupus erythematosus presenting as optic neuritis. Ann Ophthalmol.

[8] Siatkowski RM, Scott IU, Verm AM, Warn AA, Farris BK, Strominger MB, Sklar EM (2001). Optic neuropathy and chiasmopathy in the diagnosis of systemic lupus erythematosus. J Neuroophthalmol.

[9] Im CY, Kim SS, Kim HK (2002). Bilateral optic neuritis as first manifestation of systemic lupus erythematosus. Korean J Ophthalmol.

[10] Lateef A, Lim AY (2007). Case reports of transient loss of vision and systemic lupus erythematosus. Ann Acad Med Singapore.

[11] Frigui M, Frikha F, Sellemi D, Chouayakh F, Feki J, Bahloul Z (2011). Optic neuropathy as a presenting feature of systemic lupus erythematosus: two case reports and literature review. Lupus.

[12] Amigo MHL, Bárbara ECD, Ghirelli W (2012). Autoimmune optic neuropathy as the first manifestation of systemic lupus erythematosus. Rev Bras Oftalmol.

[13] Heckman AJ, Alsaad AA, Stewart MW, Maniaci MJ (2019). Acute unilateral vision loss due to optic neuropathy in a patient with systemic lupus erythematosus. Am J Med Case Rep.

[14] Galindo-Rodríguez G, Avina-Zubieta JA, Pizarro S, de León VDa, Saucedo N, Fuentes M, Lavalle C (1999). Cyclophosphamide pulse therapy in optic neuritis due to systemic lupus erythematosus: an open trial. Am J Med.

[15] Szymezak J, Ankri A, Fischer A, Darnige L (2010). Hydroxychloroquine: a new therapeutic approach to the thrombotic manifestations of antiphospholipid syndrome [Article in French]. Rev Med Interne.

[16] Tugcu B, Acar N, Coskun C, Celik S, Yigit F (2014). Nonarteritic anterior ischemic optic neuropathy as the presenting manifestation of primary antiphospholipid syndrome. Indian J Ophthalmol.

